# Thorndike's Law 2.0: Dopamine and the Regulation of Thrift

**DOI:** 10.3389/fnins.2012.00116

**Published:** 2012-08-10

**Authors:** Jeff A. Beeler

**Affiliations:** ^1^Department of Neurobiology, University of ChicagoChicago, IL, USA

**Keywords:** J, reward, reinforcement learning, explore-exploit, temporal difference, incentive-salience

## Abstract

Dopamine is widely associated with reward, motivation, and reinforcement learning. Research on dopamine has emphasized its contribution to compulsive behaviors, such as addiction and overeating, with less examination of its potential role in behavioral flexibility in normal, non-pathological states. In the study reviewed here, we investigated the effect of increased tonic dopamine in a two-lever homecage operant paradigm where the relative value of the levers was dynamic, requiring the mice to constantly monitor reward outcome and adapt their behavior. The data were fit to a temporal difference learning model that showed that mice with elevated dopamine exhibited less coupling between reward history and behavioral choice. This work suggests a way to integrate motivational and learning theories of dopamine into a single formal model where tonic dopamine regulates the expression of prior reward learning by controlling the degree to which learned reward values bias behavioral choice. Here I place these results in a broader context of dopamine's role in instrumental learning and suggest a novel hypothesis that tonic dopamine regulates thrift, the degree to which an animal needs to exploit its prior reward learning to maximize return on energy expenditure. Our data suggest that increased dopamine decreases thriftiness, facilitating energy expenditure, and permitting greater exploration. Conversely, this implies that decreased dopamine increases thriftiness, favoring the exploitation of prior reward learning, and diminishing exploration. This perspective provides a different window onto the role dopamine may play in behavioral flexibility and its failure, compulsive behavior.

## Introduction

Thorndike (1911) first articulated his **Law of Effect** that states:

“*Of several responses made to the same situation those which are accompanied or closely followed by satisfaction to the animal will, other things being equal, be more firmly connected with the situation, so that, when it recurs, they will be more likely to recur*… the greater the satisfaction, the greater the strengthening…”

In his studies, Thorndike (1911) placed cats into enclosures with trick latches. Being cats, they do not like being imposed upon and so the animals began exploring, pawing, and figuring out how to open the door. Eventually, they figure it out. Upon repeated exposures, they come to open the door quickly without engaging in preliminary, exploratory behaviors. In short, the cats learned to open the door. Cats are clever.

**Figure 1:**
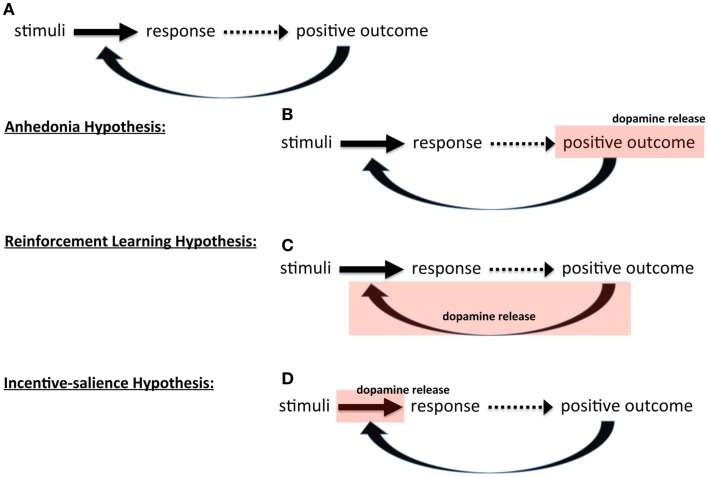
**Theories of dopamine and instrumental behavior**. Schematic showing **(A)** basic outline of instrumental, stimulus-response learning three hypothesis on the role of dopamine: **(B)** anhedonia hypothesis, **(C)** reinforcement learning hypothesis, and **(D)** incentive-salience hypothesis.

Thorndike (1911) formalized the common sense observation that “they learned to open the door” by suggesting a learning mechanism with two key elements: (a) *what is learned* – an association between stimuli in the environment and the behavior such that when those stimuli are presented, the behavior is more likely to be emitted, (b) *how learning occurs* – a positive outcome increases the strength of that association increasing its impact on future behavior. Thorndike's formulation articulates the basic concept of instrumental learning: a positive outcome following an emitted behavior (response) strengthens the association between stimuli present during the behavior and the particular response to those stimuli that yielded “satisfaction” (Figure 1A). We tend to repeat things that work out well for us.

Importantly, Thorndike's (1911) law is probabilistic: behaviors will *be more likely* to recur. What determines the likelihood of recurrence? As the quote from Thorndike suggests, he – and most investigators in the subsequent century – have focused on the magnitude of the positive outcome and the strength of the association it induces to determine how much learning controls behavior. Here we develop the idea that the degree to which prior learning biases behavior can be regulated independently of the strength of those associations. We will map this onto temporal difference reinforcement learning algorithms where the contribution of the strength of association is captured in the concept of *value*; that is, a formalization of “satisfying effect.” The degree to which these values bias behavior is represented in the concept of *temperature*. The probability of recurrence – the “likely,” then, is a function of both. Here we focus on the latter.

## Dopamine and Reward: Controversy and Consensus

Understanding the neural substrates that mediate instrumental, stimulus-response learning remains an important question in neuroscience, a question that invariably leads to dopamine and **reward**. Though widely believed that dopamine plays a role in mediating the impact of reward on behavior – often construed as the “reward system” – its precise function remains controversial. Though widely used, “reward” is an ill-defined term (Cannon, 2004; Salamone et al., 2005; Salamone, 2006; Yin et al., 2008). It is sometimes meant to refer to positive affect associated with a “satisfying effect.” At other times it is used interchangeably with “reinforcer” and yet other times as something that satisfies an appetitive need. Here we use the term broadly as synonymous with positive outcome (“satisfying effect”) without distinguishing different aspects of a positive outcome. Similarly, there has obviously been much elaboration of instrumental learning since Thorndike. For example, “stimulus-response” is widely viewed as a particular type of instrumental learning associated with habitual, automatic responding while other forms, such as action-outcome learning, represent goal-directed associative learning (Thorndike, 1911; Yin and Knowlton, 2006; Balleine and O'Doherty, 2010). Here we return to the broad umbrella provided by Thorndike in which the heart of associative learning, of all stripes, is to associate stimuli with the appropriate action or response. We will revisit finer distinctions below.

Additionally, midbrain dopamine nuclei project widely throughout the brain, including projections to the prefrontal cortex, amygdala, hippocampus, and the striatum (Haber and Knutson, 2010). However, the great bulk of investigation into dopamine function has centered primarily on its projections to the striatum, widely viewed as a key substrate of reinforcement learning and the predominant site of action in dopamine mediation of reward and motivation (Schultz, 2002; Everitt and Robbins, 2005; Balleine et al., 2007; Nicola, 2007; Belin et al., 2009; Wise, 2009; Humphries and Prescott, 2010; Ito and Doya, 2011). Thus, the following discussion of dopamine and reinforcement learning applies to dopaminergic processes within the striatum. Importantly, however, a primary function of the striatum is widely believed to be the modulation of cortical activity vis-a-vis corticostriatal loops (Alexander and Crutcher, 1990; Parent and Hazrati, 1995; Haber, 2003; Graybiel, 2005; DeLong and Wichmann, 2009; Redgrave et al., 2011), providing a substrate by which reward-based (reinforcement) associative learning can influence cortically mediated behavior (for interesting review, see Cools, 2011). The degree to which other dopamine targets may contribute to the dopamine function proposed here is beyond the scope of the current, brief review.

The different views of dopamine and reward can be mapped onto Thorndike's (1911) law, though this is not to suggest that mediating or modulating instrumental, stimulus-response learning is the sole function of dopamine. Broadly, theories of dopamine's role in reward can be classed into three perspectives.

In the first, initially proposed by Roy Wise (Wise et al., 1978; Cannon, 2004; Salamone et al., 2005; Salamone, 2006; Yin et al., 2008; Wise, 2010), dopamine itself induces pleasure; that is, when something good happens, this positive outcome releases dopamine that creates pleasure that then reinforces the behavior. In essence, in this view dopamine mediates *affect* that determines reinforcement; dopamine release *is* the positive outcome (Figure 1B). In the absence or reduction of dopamine, there is a lack of pleasure and consequently a lack of reinforcement, giving this perspective its name, the anhedonia hypothesis. This view has been widely criticized on the basis that dopamine is not, in fact, mediating pleasure or affect (Salamone et al., 1997; Berridge and Robinson, 1998).

The second broad perspective is that dopamine mediates learning itself. In this view dopamine *signals* a positive outcome that induces learning, i.e., alters the associations between stimuli and responses. There are two versions of this. One, developed by Wise (2004) from his original anhedonia hypothesis, argues that dopamine “stamps in” reinforcement; interestingly, the same term used by Thorndike (1911): dopamine is a reward hammer, fashioning synapses to conform to reward feedback. The other perspective draws upon learning theory and dynamic programming to formalize reinforcement learning as a specific algorithm called **temporal difference learning** (Sutton and Barto, 1998). In this perspective, the strength of association between stimuli and response encodes the value (expected reward) of emitting *that* behavior in response to *those* stimuli. The greater the value, the more likely the behavior is to be emitted. In these models, the emphasis is on assigning the “*correct*” value to these associations. Dopamine, then, is assigned a very specific role called a prediction error signal (Montague and Dayan, 1996; Schultz et al., 1997; Schultz, 2002). Instead of signaling at every positive outcome and “stamping in” reward, dopamine only signals when the outcome was unexpected, either more or less reward than anticipated, and thus rather than stamping in reward, it *updates* reward value. The formalism of reinforcement learning models forces the clear identification of parameters that affect the performance of the algorithm, and these parameters represent a conceptual strength of such models, which we will return to below. The key point here is that in the learning view of dopamine, dopamine serving as teaching signal (Figure 1C).

The third broad perspective is the motivational view, where dopamine is viewed as serving an activational function, invigorating goal pursuit (Robbins, 1992; Robbins and Everitt, 2007), including the widely accepted incentive-salience hypothesis (Robinson and Berridge, 1993; Berridge, 2004, 2007). In incentive-salience, the association between stimuli and a response is construed as incentive and dopamine scales that incentive up or down, dynamically regulating the degree to which learned associations influence behavior (Figure 1D). Salamone et al. (2007) have developed an alternative motivational view and argue that dopamine does not modulate reward at all; instead, dopamine energizes behavior and decreases the impact of *response cost* (i.e., effort required) on behavioral choice: “… enabling organisms to overcome obstacles or work-related response costs that separate them from significant stimuli.” Though they propose very different mechanisms, in both theories, dopamine effectively increases motivation (energy expended toward a goal), increasing the *likelihood* of reward pursuit – either by energizing behavior (reducing cost barriers) in Salamone's view or inducing “wanting” (increasing incentive) in Berridge's.

Though these different perspectives are often viewed as competing hypotheses and inspire controversy, there is nothing inherently mutually exclusive between them. It is conceivable that dopamine *could* do all these things. The challenge is to discern how these potential aspects of dopamine function may be integrated. Here we will focus on integrating the reinforcement learning and motivational views into a single framework.

## Anti-Automaton Mechanisms

The potential problem with stimulus-response learning as a mechanism controlling behavior is a lack of flexibility: organisms could become enslaved to their prior reward history, simply emitting learned, reinforced responses to stimuli. Indeed, behaviorists in the early to middle twentieth century saw principles of S-R learning as a way to shape and control human behavior (Skinner, 1948, 1971). Though both humans and animals ended up being more resistant to such control than anticipated (for an interesting historical survey of behaviorism, see Lemov, 2005), the notion of being a slave to S-R learning persists in modern neuroscience, particularly in the study of dopamine, where over-activating the dopamine system, for example through drugs of abuse (Berke and Hyman, 2000; Hyman et al., 2006) or, more recently, highly palatable food (Volkow and Wise, 2005; Avena et al., 2008; Kenny, 2010), establishes associations that results in compulsive behavior that escapes rational, executive control (Everitt and Robbins, 2005; Kalivas and O'Brien, 2008). Berridge (2007) has argued against a habit-based “automaton” view of addiction, noting that addicts can be highly flexible and inventive in their pursuit of drugs. Nonetheless, even in his theory, learned incentives induce increased “wanting,” generating compulsive behavior: addicts become slaves to “wanting” rather than habit.

The primary check on slavish S-R responding is generally believed to be a cognitive, deliberative system that exerts executive control, inhibiting and intervening in the automatic responses that might otherwise be generated by reinforced stimulus-response associations. This is reflected in theories of addiction where not only do drug-reinforced S-R associations predominate in an addict's behavioral control, but there is a concomitant failure of executive control (Hyman et al., 2006; Kalivas, 2008; Koob and Volkow, 2010). Executive control can be cast in several forms. There is traditional, hierarchical executive control, which is associated primarily with the prefrontal cortex, where a higher level of cognitive function exerts inhibitory control over the expression of more rudimentary associative learning, bringing reward pursuit under goal-directed control. In addition, there are “two-system” theories of behavioral control. In these models, S-R learning comprises one system that generates habitual, automatic responding while another, potentially competitive system, generates goal-directed behavior. For example, in a widely adopted learning typology, S-R learning is viewed as habitual and automatic, and associated with the dorsolateral striatum, while action-outcome (A-O) learning provides an associative substrate for goal-directed behavior, associated with the dorsomedial striatum (Yin and Knowlton, 2006; Balleine and O'Doherty, 2010). There is a roughly parallel version of this cast in computational terms (Daw et al., 2005) where: (a) S-R habit arises from model-free, cached values (i.e., the value of stimuli and actions represented simply as a weight without any representation of how that value arises) and (b) goal-directed behavior arises from a model-based system where learned associations represent a model of the world (i.e., the animal can scan forward/backward in a “model tree” and calculate the relative predicted values of different behaviors based on this model rather than a “mysterious” cached value). However, insofar as S-R learning is an evolutionarily old form of learning, adaptive fitness may have required that it evolve, *within the system*, a mechanism that prevents an organism from becoming a slavish automaton to arbitrary experiences of reward.

## Explore-Exploit: Deviating from Strict S-R Control

Even for the simplest of organisms (or a computer model), adaptation requires a degree of freedom from responding strictly controlled by learned S-R associations. Always choosing the response that has, in the past, yielded the greatest reward – known as a greedy strategy – is insufficiently flexible. Reward is essentially information about the environment. This information, in turn, assigns values to stimuli, actions, and their associations, which represents an understanding of the world. The challenge for an organism is in arriving at the best understanding of the world: how does an organism know that it “got it right” or that the environment has not changed recently? Thus, new information can be valuable (Behrens et al., 2007), even if it is not directly rewarding (but see Kakade and Dayan, 2002).

The **explore-exploit dilemma** (Sutton and Barto, 1998), poses the question of how the animal is to balance fully maximizing its reward based on its current knowledge of the world, exploitation, against obtaining new knowledge, exploration (see Cohen et al., 2007 for review; Daw et al., 2006 for human study). An example will help from Dayan (2001). Imagine a bee foraging between two colors of flowers where the yield of nectar for all flowers is probabilistic but the average yield is higher (let us say double) in blue rather than yellow flowers. A bee that first lands on a low yield yellow flower would be rewarded. If that bee were to maximally exploit that reward information, it would continue to return to yellow flowers, be further reinforced, resulting in a strategy of only selecting yellow flowers. In this scenario, the bee has never has sufficiently sampled its environment and learned that blue flowers yield twice as much nectar. Of course, the bee could have landed on the blue flower first. Though fortuitous for the bee, a greedy strategy is still likely to be maladaptive if the environment is changing, as most environments do. For example, if during spring different growing patterns result in a shift such that later in spring the yellow flowers have higher yield, the bee that only selects blue flowers will suffer a disadvantage. Thus, an organism needs to be able to regulate how much reward history biases behavioral choice, allowing non-greedy exploration: sampling and updating of knowledge of the environment.

## Mapping Temporal Difference Learning onto Thorndike

**Figure 2:**
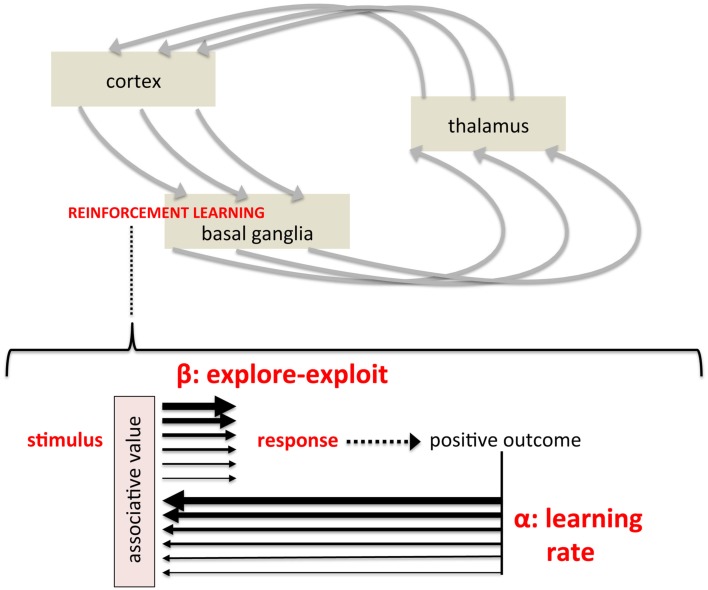
**Mapping formal parameters of reinforcement learning models onto instrumental, stimulus-response learning**. (Top) Simplified depiction of cortico-basal ganglia-thalamo-cortical circuits believed to modulate cortical activity and action selection, representing a pathway by which striatum-based reinforcement learning influences behavior. (Bottom) A schematic showing the two key parameters of temporal difference models within a simple stimulus-response diagram. The light red box labeled “associative value” represents the synaptic strength, construed as “value” in computational models, linking a particular stimulus with a particular response. The learning rate reflects dopamine's modulation of synaptic plasticity, regulating the degree to which outcomes alter learned values (represented by thickness of black arrows). The explore-exploit parameter reflects the degree to which an established value biases the subsequent response (again represented by arrow thickness), reflecting dopamine's modulation of responsiveness of striatal projection neurons to afferent activity (i.e., a gain mechanism).

In reinforcement learning algorithms, stimuli and actions are associated with values (V) that arise from accumulating reward experience. More rewarding stimuli and actions will obtain greater value. These values, in term, bias action choices in favor of more rewarding options. The values are updated with new reward experience. The striatum has been implicated as a primary substrate for reinforcement learning where it is believed to play a role in action selection (Mogenson et al., 1980; Mink, 1996; Redgrave et al., 1999) by modulating cortical activity vis-a-vis corticostriatal-thalamo-cortical loops (Figure 2, top). Dopamine can alter corticostriatal throughput, and presumably action selection, through two mechanisms (Beeler, 2011; Beeler et al., 2012b): (1) direct modulation of striatal projection neuron activity and responsiveness to input (medium spiny neurons) and (2) indirectly through its regulation of corticostriatal plasticity, altering the strength of synaptic transmission as a consequence of experience. These two regulatory mechanisms of dopamine in corticostriatal throughput represent potential mechanisms underlying dopamine modulation of two different aspects of reinforcement learning.

Temporal difference models have two critical parameters that control how rewarding outcomes shape behavioral choice. The first is the **learning rate**, commonly called *alpha*, which controls the rate at which new information updates established values – learning *about* “satisfying effects.” With a high learning rate, for example, very recent experience has a great impact on values. A low learning rate, in contrast, preferentially weights cumulative experience. This parameter is central to the relationship between reward and reinforcement efficacy; that is, the degree of reinforcement induced by reward (Figure 2, bottom), though obviously the nature and magnitude of the reward itself is independent of learning rate. The other parameter, commonly referred to as *beta* or the “inverse **temperature**,” controls the *degree* to which established values actually biases behavioral choice (Sutton and Barto, 1998; Dayan, 2001; Daw et al., 2006). A high beta, for example, would mean that behavior is highly determined by established values, or “greedy”; a low beta, in contrast, means that behavior may deviate from what would be dictated by established values. At “maximal” beta, behavior would approach maximally greedy and behavioral choice would be ruled by prior experience. At “minimal” beta, behavior would relatively independent of prior experience, as if learning had not occurred. By controlling the degree to which learned associations and established values determine behavioral choice, this parameter can modulate the “likely” in “more likely to recur in the future” (Figure 2). In short, Thorndike (1911) states that obtaining a “satisfying effect” – rewarding outcome – will increase the probability of that behavior in the future. However, that increased probability can be considered to be the result of two factors: (1) the magnitude and reinforcement efficacy of the reward itself, or more precisely the subsequent strength of association and (2) the degree to which learned associations bias behavioral choice. Computational reinforcement learning models formalize these two aspects of reinforcement learning, the translation of “satisfying effects” to behavioral choice, as these two critical parameters, alpha and beta, the learning/update rate, and the balance between exploration and exploitation.

## Mapping Phasic and Tonic Dopamine onto Temporal Difference Learning

Dopamine is widely believed to operate in two modes, *phasic* and *tonic* (Grace et al., 2007), but see Joshua et al. (2009), Owesson-White et al. (2012). **Phasic dopamine** activity consists of short burst of high-frequency spikes while tonic activity consists of irregular, basal spike activity at approximately 4–5 Hz. The role of dopamine as prediction error signal in temporal difference learning models is associated with phasic firing (and pausing) with millisecond temporal resolution appropriate to signal discrete events, such as cues and rewards (Schultz et al., 1997; Schultz, 2002; Stuber et al., 2005; Day et al., 2007; Flagel et al., 2010; Gan et al., 2010). In contrast, tonic activity, investigated primarily through pharmacological and genetic methods, is more widely associated with motivational views of dopamine function and acts across a broader temporal span. The putative roles of dopamine in mediating reinforcement learning and modulating motivation, often associated with phasic and **tonic dopamine**, respectively, have not been reconciled into a single, unified theory. Though the role of phasic dopamine in reinforcement learning has been extensively modeled using temporal difference algorithms, there has been little examination of the potential role of *tonic* dopamine in these models. The incentive-salience perspective on dopamine suggests that dopamine scales the impact of learned reward values (i.e., incentive) on behavior, suggesting a potential way to integrate tonic dopamine and its putative motivational effects into reinforcement learning models as a mechanism regulating the beta, or explore-exploit, parameter (Beeler et al., 2010, see Zhang et al., 2009 for an alternative approach).

## Dopamine and Beta: Regulating How Value Biases Choice

Given that dopamine is widely associated with enhanced pursuit of reward and believed to underlie compulsive behavioral disorders such as addiction, we asked whether increased tonic dopamine would impair behavioral flexibility (Beeler et al., 2010); that is, make mice more greedy such that their behavioral choices were more dictated by prior reward experience. This question has two components: (1) does *tonic* dopamine affect beta, the degree to which reward learning biases behavior and (2) if so, what is the direction of dopamine's modulation of beta? Prior literature would suggest that increased tonic dopamine would increase the impact of reward on behavioral choice. Thus, we might expect that hyperdopaminergic mice will show increased beta and be more controlled by reward history.

To test this we used a homecage behavioral flexibility paradigm. Mice were singly housed in cages equipped with two operant levers and a pellet dispenser. There was no food restriction but all food had to be acquired through lever pressing. Both levers yielded 20 mg pellets of food, but with different lever press requirements such that one was always “cheap” (FR20, fixed ratio schedule requiring exactly 20 presses per pellet) and one was always expensive where the cost incremented across the experiment (FR20-250). Which lever was which, however, randomly changed every 20–40 min. As a consequence, the mice had to monitor their on-going reward and constantly update the value of each lever based on recent returns. To assess the role of tonic dopamine, we used mice with a knock-down in the dopamine transporter (DATkd) that results in increased extracellular dopamine and an increased rate of tonic activity (Zhuang et al., 2001) while patterns of phasic, burst activity are unchanged (Cagniard et al., 2006).

Behaviorally, we observed what would be expected: the DATkd mice work harder and spend more time pressing the expensive lever. In this situation, however, they do not gain significantly greater reward; they just work harder. One potential explanation would be that the DAT mice have impaired learning and do not update the value of each lever in the same way that wild-type mice do. When we examined their behavior immediately surrounding switches between which lever was expensive and inexpensive, however, we observe identical patterns in the two genotypes: both groups recognize the change, receive reward on the now cheap lever but then nonetheless return to the previously inexpensive lever for a short time before gradually shifting their effort toward the now inexpensive lever. The difference between the groups does not lie in their behavior around switches but rather during the periods of stability between these switches where the wild-type preferentially distribute their pressing toward the inexpensive lever while the DATkd distribute their effort equally to both levers. This suggests that the DATkd are adopting an alternative behavioral strategy.

To formally assess how the two genotypes are using on-going reward information, we fit the data to a temporal difference learning algorithm (Daw and Dayan, 2004; Corrado et al., 2005; Lau and Glimcher, 2005) and modeled the data on a lever press by lever press basis. We observed no difference between genotypes in the alpha, or learning rate parameter, consistent with previous studies of the DATkd showing no learning impairments (Cagniard et al., 2006; Yin et al., 2006; Beeler et al., 2010) and their behavior around lever switches described above. The DATkd appear to learn about and update reward information normally. In contrast, we observed a difference in the beta parameters between the two groups where the DATkd exhibit a lower beta; that is, there was *less* coupling between reward history and their behavioral choices. From this we draw two primary conclusions. First, that tonic dopamine can modulate the inverse temperature, or explore-exploit parameter in a TD learning model, suggesting that tonic dopamine plays a complementary role to phasic dopamine, where the latter modulates learning and updating values while the former scales the degree to which those values bias behavioral choice. Second, we observe that increased dopamine *decreases* the impact of reward value on behavioral choice: that is, elevated dopamine favors *exploration* rather than *exploitation*. Subsequent modeling work by Humphries et al. (2012) supports these observations and elaborates the potential complexity of dopamine modulation of the trade-off between exploration and exploitation.

The observation that tonic dopamine can modulate how much learned values bias behavior is consistent with the incentive-salience hypothesis where dopamine scales incentive values that drive reward pursuit (Cagniard et al., 2006; Berridge, 2007). However, rather than *increasing* the impact of learned values on choice behavior, as predicted by the incentive-salience hypothesis, increased dopamine, in these studies, *diminishes* the coupling of reward and choice.

## Ruling Out Other Explanations: Cost Sensitivity and Perseveration

Increased dopamine has been associated with perseveration and stereotypy, making it possible that the DATkd “get stuck” motorically or even decision-wise. The data rule this out. In the model, a “last lever pressed” factor was included to capture the degree of perseveration, i.e., that the greatest factor determining their lever choice is simply the lever they last pressed. There were no differences between genotypes on this factor, indicating that the DATkd mice are not simply perseverating.

Alternatively, Salamone et al. (2007) have long argued that dopamine *energizes* behavior, increasing the amount of effort an animal will expend in pursuit of a goal. In this view, increased dopamine does not enhance the incentive for reward but, rather, diminishes sensitivity to cost associated with procuring reward. Thus, it is possible that the DATkd mice “simply did not care” about the cost. The data argue against this. In a variant of the paradigm where the expensive and inexpensive levers did *not* switch, the two genotypes were indistinguishable and the DATkd clearly favored the inexpensive lever. In a subsequent study (Beeler et al., 2012a) with escalating response costs to obtain food, the DATkd showed the same elasticity of demand – the degree to which consumption adjusts in response to escalating cost (Hursh and Silberberg, 2008) – as wild-type, again suggesting that the DATkd mice are not insensitive to cost. Nonetheless, as Salamone suggests, dopamine does appear to energize their behavior. If this is not due increased incentive (i.e., increased exploitation of reward value information) nor to decreased sensitivity to cost, how then is their behavior being “energized?”

Before proceeding, it is important to note that our results show a *decrease in exploitation*. Though exploration and exploitation are often construed as occurring along a continuum, and *behaviorally* an increase in one necessitates a decrease in the other, at the level of neural processes, exploitation and exploration may represent two (or more) interacting processes mediated by different neural substrates. Thus, in our studies, enhanced dopamine resulted in decreased exploitation (decreased coupling between reward history and choice), *allowing* exploration. The degree to which the behavioral strategy of equally sampling two familiar levers represents exploration is unclear. In the remainder of the review, we argue that dopamine favors exploration; however, we view this primarily as *permissive*. We remain agnostic as to what mechanisms *direct* exploration (see Cohen et al., 2007). Critically, the beta parameter, though generally viewed as regulating explore-exploit, also captures noise in the model, including factors affecting the process being modeled but not included in the model (Nassar and Gold, 2010). This means that “exploration” may actually reflect some other, unaccounted for, process or factor. Here, we propose such a factor: energy availability, signaled by dopamine, regulates *thrift*, or the degree to which prior reward information needs to be maximally exploited. In short, we construe the dopaminergic regulation of beta as the regulation of **thrift**.

## Adapting Behavior to the Economic Environment: Regulating Thrift

A central function of reinforcement learning is to maximize reward (Sutton and Barto, 1998; Dayan, 2001). As noted above, however, this requires balancing the greedy exploitation of prior knowledge and exploring the environment, ensuring one's knowledge of the environment is accurate and updated. The best balance between exploration and exploitation, however, is contingent on the environment, both its stability and its economics. The former is reflected in the certainty an animal can attribute to its knowledge (see Daw et al., 2005; Yu and Dayan, 2005; Rushworth and Behrens, 2008), an important topic outside the scope of the current discussion. The latter is reflected in the availability and cost of reward. In the context of foraging, if food is reliably and readily available, there is no advantage to being frugal. Energy spent exploring will increase the animal's knowledge of its environment and facilitate adaptation. In contrast, if food is scarce and costly, exploration should be kept to a minimum to conserve energy and exploit the limited available food sources.

Niv et al. (2007) proposed that tonic dopamine regulates *vigor* of behavior by encoding the average reward over time. They proposed that higher tonic dopamine would signal a rich reward environment and represent the opportunity cost associated with inactivity, the “cost of sloth.” An alternative take on this same notion would suggest, as Niv proposes, that tonic dopamine represents the average reward history – from our view, specifically energy availability – but that a rich environment would favor *exploration* and energy expenditure rather than exploitation of reward information. That is, if the environment is rich, opportunity costs are not important: there is plenty to go around, one should expend energy and explore. Activity may be increased but less strictly coupled to reward. What is being adapted, in this view, is not reward pursuit but the expenditure of energy relative to the prevailing energy economy (Beeler et al., 2012b). Our data suggest a relaxation of thrift, that is, decreased exploitation, *permissive* of exploration. How this energy “freed from the Law of Effect” is directed behaviorally is a different question, beyond the scope of this review.

## The Role of Explore-Exploit Regulation: Dopamine Modulates Thriftiness

**Figure 3:**
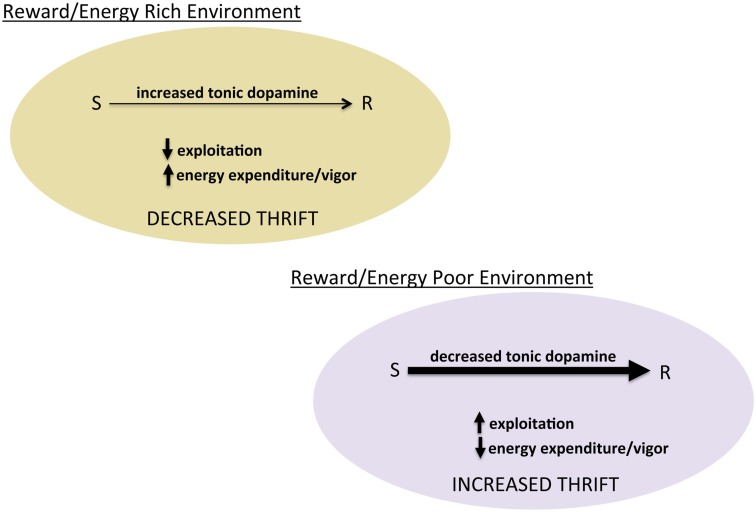
**Tonic dopamine and the balance between exploration and exploitation**. Schematic of hypothesized role of tonic dopamine in mediating thrift in energy expenditure and reward pursuit through regulating the degree to which prior reward learning and value biases behavioral choice.

The idea that dopamine regulates energy expenditure, though not central to the reward-centric view of dopamine, is consistent with decades of literature showing that dopamine modulates general activity levels, exemplified by psychostimulants. What is not intuitive is the relationship between dopamine's modulation of activity as a generalized phenomenon and its putative regulation of the balance between exploration and exploitation. We suggest that in a given energy economy, the animal faces two fundamental questions: (1) how much energy do I have to expend and (2) how thrifty do I need to be in using available energy (put another way, how carefully should I distribute that energy). The balance between exploration and exploitation can be thought of, energetically, as regulating thriftiness. In a reward/energy rich environment, there is no need to conserve or be frugal with energy expenditure. In such an environment, increasing exploration and energy expended in foraging (or other behaviors) represents an adaptive decrease in thriftiness. In contrast, in reward/energy poor environments, frugality in energy expenditure is critical. Thus, in environments with a low average rate of reward (especially energy poor), exploiting prior experience to increase thriftiness and maximize return on expenditures is adaptive (Figure 3). From this perspective, learned reward value, or incentive, can be thought of as *guidelines* for managing the *distribution* of energy resources. The degree to which these incentives/values control behavior, however, depends upon the balance between exploration and exploitation, modulated by the same dopamine system believed to teach reward values in the first place. Insofar as the alpha and beta parameters in reinforcement learning algorithms capture something essential about this type of learning and its control over actions, a dopamine system that regulates both represents an elegant evolutionary solution to learning from experience.

Importantly, this is not to suggest that dopamine is a sole regulator of the balance between exploration and exploitation. Elegant work by Aston-Jones and Cohen (2005) has suggested that the norepinephrine system may contribute importantly to regulating the balance between explore-exploit as well. Undoubtedly multiple systems regulate this balance, each contributing a different function; for example, regulating explore/exploit as a function of uncertainty or utility (Cohen et al., 2007). Here we suggest dopamine regulation of beta may be construed as *thrift*, the degree to which an organism needs to exploit its prior knowledge to maximize return on energy investment.

## Returning to Thorndike: Freedom has its Limits

The foregoing discussion suggests that organisms need not become slavish automatons responding to arbitrary reward history, even without elaborate cognitive, executive controls. Viewing Thorndike's (1911) basic insight into stimulus-response learning from the point of view of temporal difference learning models provides an anti-automaton mechanism within S-R learning itself: the ability to adjust the *degree* to which one's behavior is ruled by the law of effect. This freedom, however, is not arbitrary or absolute. Instead, it arises in response to the richness of the environment – fundamentally, the energy environment – which we suggest is conveyed by tonic dopamine.

Most views of dopamine and reward argue that dopamine increases the yoke between reward and behavioral choice. The perspective outlined here suggests that dopamine regulates the yoke between energy availability, or the richness of the environment, and the thriftiness of behavioral energy expenditure. Ultimately, this view may help resolve paradoxes in the dopamine field. For example, it is paradoxical that obesity has been associated with *reduced* dopamine function (Di Chiara et al., 1998; Wang et al., 2001; Davis et al., 2008; Geiger et al., 2008, 2009; Li et al., 2009; Vucetic and Reyes, 2010), giving rise to the “reward deficiency hypothesis” (Wang et al., 2001; Geiger et al., 2009; Kenny, 2010). An alternative explanation, from the current perspective, is that reduced dopamine increases thriftiness, favoring exploitation of reward information, energy intake, and conservation of energy expenditure, an obvious recipe for obesity (Beeler et al., 2012b). Within addiction, the relative contribution of hyper- and hypo-dopamine function, associated with sensitization theories (Robinson and Berridge, 2001) and the reward deficiency hypothesis (Blum et al., 2000, 2011), respectively, remains an active area of investigation (Koob and Volkow, 2010). From the current perspective, a reduction in dopamine tone, either acutely or chronically over time, would result in thrifty exploitation of established reward value – a shift toward greedy, S-R control – which for an addict would mean focusing energy on drug pursuit. This view highlights not only how dopamine may facilitate drug-seeking, but how the capacity to seek and learn from other sources of reward – the ability to explore – is diminished, locking addicts in an ever diminishing world of limited reward to which they respond increasingly desperately and slavishly as their behavior becomes increasingly dominated by a distorted Law of Effect.

## Conflict of Interest Statement

The author declares that the research was conducted in the absence of any commercial or financial relationships that could be construed as a potential conflict of interest.

## Key Concept

Law of effect: Early description of instrumental learning where a positive outcome increases the likelihood of a behavior being repeated in the future under similar circumstances.
Reward: Here construed simply as “positive outcome” and taken as synonymous with reinforcer. More precise definitions have been proposed but are not germane to the present discussion.
Temporal difference learning: A class of algorithms for implementing supervised reinforcement learning in which differences in predictions across successive time steps are used to update preceding predictions. For example, a prediction of expected reward at time *t* is compared with actual reward plus predicted future reward at time *t* + 1. The difference between *t* + 1 reward/prediction and the prediction at t is used to update the prediction at time *t*.
Explore-exploit dilemma: An expression coined to capture the trade-off between fully exploiting prior reward learning by always choosing the best option (exploit) and sampling less valuable options to obtain further information about the environment (explore). In TD models there are multiple potential strategies for managing exploration-exploitation, but the neural mechanisms controlling this trade-off remain poorly understood.
Learning rate: The degree to which new reward information updates established values, controlling the relative contribution between new and old information; by providing a temporal window of integration, controls the rate of learning and forgetting.
Temperature: Amount of “randomness” in a system; in TD models, determines the degree to which established values bias behavioral choice (usually the *inverse* temperature).
Phasic dopamine: Short, high-frequency bursts of spikes in dopamine cells associated with prediction error signals in reinforcement (temporal difference) learning theories of dopamine.
Tonic dopamine: Irregular, low frequency basal firing activity of dopamine cells; extracellular dopamine concentration or dopamine “tone.” Note, extracellular dopamine concentrations may be the consequence of both tonic and phasic dopamine activity.
Thrift: The degree to which behavioral choice is biased toward maximizing the return on energy expenditure, i.e., minimizing effort and maximizing reward.
